# Poleward expansion of common snook *Centropomus undecimalis* in the northeastern Gulf of Mexico and future research needs

**DOI:** 10.1371/journal.pone.0234083

**Published:** 2020-06-22

**Authors:** Caleb H. Purtlebaugh, Charles W. Martin, Micheal S. Allen

**Affiliations:** 1 Fish and Wildlife Research Institute, Florida Fish and Wildlife Conservation Commission, Senator George Kirkpatrick Marine Laboratory, Cedar Key, Florida, United States of America; 2 Fisheries and Aquatic Sciences Program, Institute of Food and Agricultural Sciences, Nature Coast Biological Station, University of Florida, Cedar Key, Florida, United States of America; Department of Agriculture, Water and the Environment, AUSTRALIA

## Abstract

Globally, rising temperatures have resulted in numerous examples of poleward shifts in species distribution patterns with accompanying changes in community structure and ecosystem processes. In the Gulf of Mexico, higher mean temperatures and less frequent winter freezes have led to the expansion of tropics-associated marine organisms. Our objectives were to quantify changing environmental conditions and the poleward expansion of the common snook *Centropomus undecimalis* into the Cedar Keys area of Florida, USA (29 deg N). The snook is an economically and recreationally important sport fish found from southern Brazil to south Florida. Cedar Key and the Lower Suwannee River are north of the snook’s historically documented range, likely due to lethal water temperatures during winter. Using data from a long-term monitoring program, we report an increase in catches of snook in this area since 2007. The spatial and temporal expansion of the species began with adult fish in 2007. By 2018, snook of all sizes were found in the region, and we found strong evidence of local reproduction during 2016–2018. The locations of nursery habitat and winter thermal refuges (e.g., freshwater springs) need to be identified and have implications for land-use policy and minimum-flow regulations for rivers. The arrival of the snook in the northern Gulf of Mexico could affect food web ecology and habitat interactions among estuarine predators, and future studies should evaluate snook’s food habits and competitive interactions with resident fishes in this expanded range. Our study provides an example of how species range expansions due to changing temperatures should result in new research priorities to evaluate impacts of climate change on coastal systems.

## Introduction

Increases in temperature have influenced the physiology, phenology, and geographic ranges of organisms [1,2] and, with models predicting an additional increase of 2.4–6.4°C over the next 100 years [3], is expected to continue. These changes can therefore influence spatial habitat use and trophic dynamic processes in food webs [4–6], and rising temperatures have been implicated in the northward spread of a variety of organisms in the Gulf of Mexico (hereafter referred to as Gulf). The lack of heavy frosts has allowed a number of tropical/subtropical species to extend into and persist in the northernmost reaches of the Gulf. For example, black mangrove *Avicennia germinans* have become common throughout the Gulf [7–10], including Texas [11], Louisiana [12], Mississippi [13], and Florida [14]. Both red and white mangroves (*Rhizophora mangle* and *Languncularia racemosa*, respectively) are now common in Florida’s Big Bend region near Cedar Key (author’s, personal observation). Warmwater corals not previously observed in the northern Gulf, such as *Acropora palmata*, have been observed [15], and green turtles (*Chelonia mydas*) have become more abundant in the northeastern Gulf [16]. Satellite telemetry and long-term data from sighting networks also indicate that the West Indian manatee *Trichechus manatus* has increased in prevalence along northern Gulf states [17,18]. Comparisons of seagrass-associated fishes in Louisiana, Mississippi, Alabama, and northwest Florida from the 1970s to 2006–2007 indicated increased abundance of tropical and subtropical fishes (e.g., yellowtail snapper *Ocyurus chrysurus*; sergeant major *Abudefduf saxatilis*; and stoplight and emerald parrotfishes *Sparisoma viride* and *Nicholsina usta*, respectively), among others [19].

**Fig 1:**
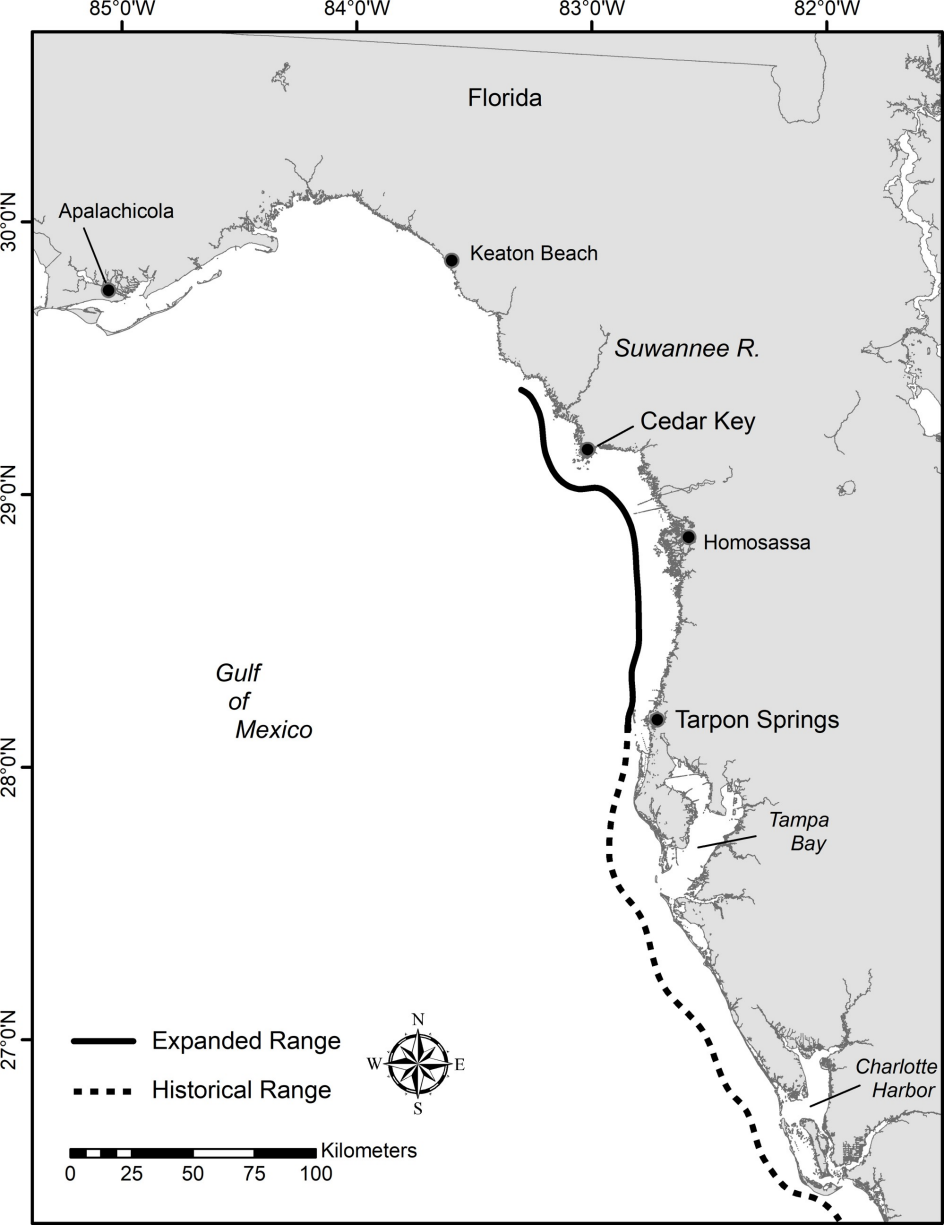
Map of historic and expanded range of snook on the Gulf coast of Florida.

Quantifying the spatial and temporal expansion of organisms into higher latitudes is important in understanding how climate change is affecting food webs and fisheries. Here, we describe the northward range expansion of common snook (*Centropomus undecimalis*, hereafter referred to as snook) into the Cedar Key and Lower Suwannee River region (Fig 1). Snook are prized game fish and support an economically important recreational fishery [20,21] in southern Gulf waters. Snook are stenothermic and highly sensitive to cold temperatures; the first sign of cold stress is the cessation of feeding, followed by loss of equilibrium and death [22–25]. We hypothesize that the combination of milder winters and warmer summers has promoted the expansion of a Florida snook population into northerneastern Gulf waters. A recent study shows a similar range expansion for snook in the western Gulf [26]. We sought to quantify the changes in snook catches using a long-term standardized sampling program, to explore the spatial extent and temporal changes in size structure, assess evidence for local reproduction, and identify future research needs for an expanding range of a subtropical apex predator in the Gulf.

## Methods

### Ethics statement

No specific permission for sampling was required, as sampling was conducted by the Florida Fish and Wildlife Conservation Commission’s Fish and Wildlife Research Institute. However, every effort was made to reduce stress and not harm captured fish, before releasing. No protected species were sampled.

### Study site

The Suwannee River lies in the northern region of the Florida Gulf Coast and is one of the largest undammed rivers in the eastern United States (Fig 1). At its mouth lies the Cedar Keys National Wildlife Refuge and the Big Bend Seagrass Aquatic Preserve. This area of Florida, known as the Nature Coast, has received high priority for conservation in the northern Gulf based on the presence of imperiled species and their habitats [27]. The region around the Suwannee River estuary also represents an ecotone between poleward-shifting mangroves and temperate salt marshes [8,14] and in recent years has seen an increase in occurrence of tropical species such as red mangrove, roseate spoonbill *Platalea ajaja*, and snook, the focus of this study. The study area contains a diverse mix of estuarine, river, and tidal creek habitats which includes extensive seagrass beds, oyster reefs, mangroves, salt marshes, and unvegetated bottom that support valuable recreational and commercial fisheries.

### Environmental conditions

To evaluate temperature changes in the region, we used a publicly available data set from a stationary weather instrument maintained and located in Cedar Key (National Oceanic and Atmospheric Administration (NOAA) gauge CDRF1, http://www.ndbc.noaa.gov/station_page.php?station=cdrf1). We attempted to obtain a long-term time series of water temperature data, but data gaps precluded use of water temperatures. Therefore, we used air temperature data from NOAA gauge CDRF-1 instead of water temperature because the use of fixed measurements taken at a stationary location reduces variability and facilitates more consistent comparisons, and data from the site had fewer missing points than data from other sources. Finally, because the water in the larger sampling region is so shallow (<2m), water and air temperatures are likely to be similar [14,28]. We analyzed air temperature for instances of warm weather conditions by using linear regression to assess the relationship between the number of days in each year that temperature reached < 12°C (the lower lethal limit for snook [22,24]). We included 18 years of data in this analysis, dating back to 2000, when the first snook in the area was documented by fishery-independent monitoring.

### Field sampling

**Fig 2:**
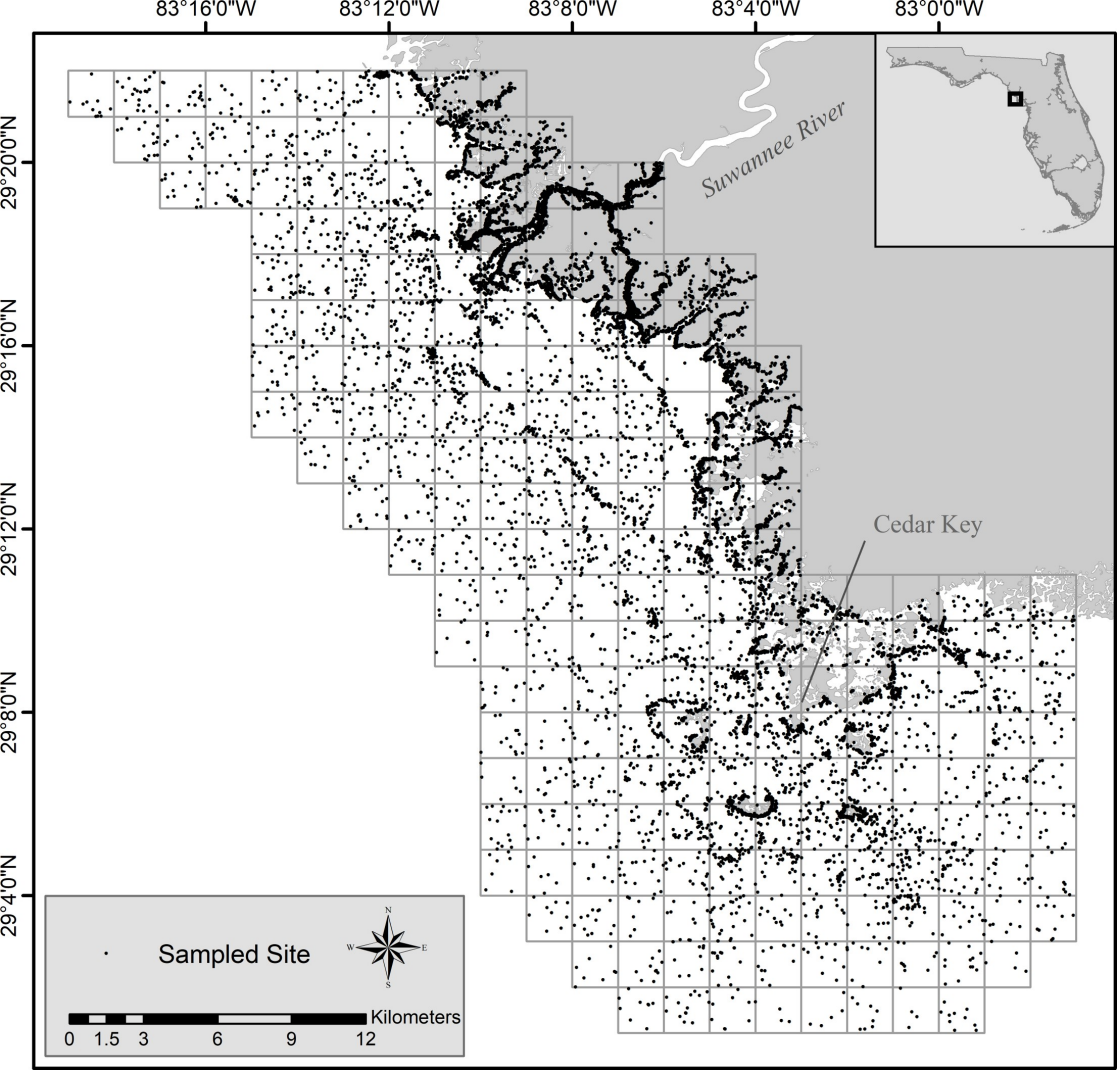
Map of study area, showing all randomly selected sample sites, 1997–2018. The grid system represents the Fisheries Independent Monitoring program’s universe.

The Florida Fish and Wildlife Conservation Commission’s Fisheries Independent Monitoring program conducted monthly standardized stratified-random sampling in the Suwannee River estuary during 1997–2018 (Fig 2). This program uses multiple gear types, including a 183-m haul seine, a 21.3-m seine, and a 6.1-m otter trawl, to collect data on various life-history stages of fishes and selected invertebrates from a variety of habitats. Detailed descriptions of site selection and standardized sampling techniques can be found in two peer-reviewed journals and a program data summary report [29–31]. In summary, the estuary was divided into geographic and logistical zones. Zones were further divided into a cartographic grid of cells measuring 1-min latitude × 1-min longitude; cells were randomly selected for sampling. Sampling cells were stratified by habitat and depth, thereby identifying the gear type and deployment technique best suited in those areas. The 21.3-m seine and the 6.1-m otter trawl targeted primarily age-0 and juvenile fishes from different depths; the 21.3-m seine sampled shallow water (≤1.8 m) along shorelines and in open-water habitat (≤1.5 m), whereas a 6.1-m otter trawl sampled relatively deep water (1.8–7.6 m). The 183-m haul seine targeted subadult and adult fish along shorelines in water depths ≤2.5 m [30]. All gear types and associated techniques were standardized with regard to amount of area fished, by following standardized sampling procedures. Effort among gear types and deployment techniques was roughly proportional to the available habitat. Sampling gear and effort are summarized in Table 1.

**Table 1:** Summary of snook collected by gear type, location, and effort in the Suwannee River estuary, Florida. Standard length = SL.

Gear	Location	Years	Effort (hauls yr^−1^)	No. fish	% Total snook catch	Min. SL	Max. SL
**183 × 2.5-m haul seine**	bay	1997	95	0	0	.	.
		1998	120	0	0	.	.
		1999–2018	192	610	97.6	105	885
			***Subtotal***	***610***	***97*.*6***		
**21.3 x 1.8-m seine**	bay	1997	216	0	0	.	.
		1998–2018	252	1	0.2	220	220
	tidal creeks	1997	72	0	0	.	.
		1998–2018	108	10	1.6	53	133
	Suwannee River	2001	55	0	0	.	.
		2002–2018	60	3	0.5	187	376
			***Subtotal***	***14***	***2*.*3***		
**6.1-m otter trawl**	bay	1997	230	0	0	.	.
		1998–2000	0	0	0	.	.
		2001	110	0	0	.	.
		2002–2018	120	0	0	.	.
	Suwannee River	2001	55	0	0	.	.
		2002–2018	60	1	0.2	331	331
			***Subtotal***	***1***	***0*.*2***		

Location included the estuary (bay), tidal creeks, and Lower Suwannee River (Suwannee River).

### Data analysis

During 1997–2018, sampling effort and locations remained nearly constant. Thus, we combined catches from all gear types each year to quantify the spatial and temporal expansion of snook. Before 2002, changes were made regarding effort and sampling location (Table 1); for example, the Lower Suwannee River was added to the sampling universe in 2001. These changes in effort and location, however, were made before snook appeared in our data, except for one snook captured in a 183-m haul seine in 2000. Sampling effort for all gear types did not change during 2002–2018. Further, >97% of all snook catches occurred in the 183-m haul seine set along shorelines, and total fishing effort using the haul seine was constant during 1999–2018. Therefore, we amalgamated all the catches of snook to evaluate temporal trends in snook catches through the time series.

We evaluated the evidence for increasing temporal trends in snook catches using Akaike information criterion (AIC) model selection [32]. The time series of snook catches was fitted to an exponential model *C* = *a×exp*^b*(*Year*)^ using maximum likelihood and a lognormal distribution, where *a* is the intercept parameter and *b* is the exponent parameter. The exponential model was compared to an intercept-only model fitted with a lognormal distribution, and AIC model selection was used to evaluate the relative credibility of each model [32]. The delta AIC values were computed between the two candidate models, and values greater than 10 are considered to have negligible credibility relative to the lowest AIC model [32].

We characterized changes in spatial distribution and quantified temporal extent of snook catches, changes in size distribution over time, evidence that snook were reproducing locally, and assessed seasonality. The spatial distribution of snook catches was plotted using GPS locations at each sample site. We then assessed the spatial location of catches through time to quantify the spatial expansion in the catches. The size structure of snook was plotted across time to quantify changes in the length distribution, show how size distribution changed throughout the time series, including presense of age-0 fish (<100 mm standard length [SL]) and subsequent juvenile fish (100 to 300 mm SL). Mean monthly catches, all years combined, were plotted with mean water temperature taken during each net deployment by month to evaluate seasonal patterns.

We used the same 22 years of data to assess trends in the frequency of occurrence of snook, black mangrove, and red mangrove. Occurrence of mangroves was recorded at each seine haul, allowing us to quantify the frequency of occurrence through time. Only data from the 183-m haul seine were used for this analysis, which sampled only shoreline areas and captured the majority (>97%) of snook in this study. We also plotted the frequency of occurrence of shore types coded as black mangroves or red mangroves during 183-m haul sampling, since mangroves have been shown to be important habitat for snook [29] and the climatic factors affecting both snook and mangroves appear to be similar [8, 33].

## Results

### Environmental conditions

**Fig 3:**
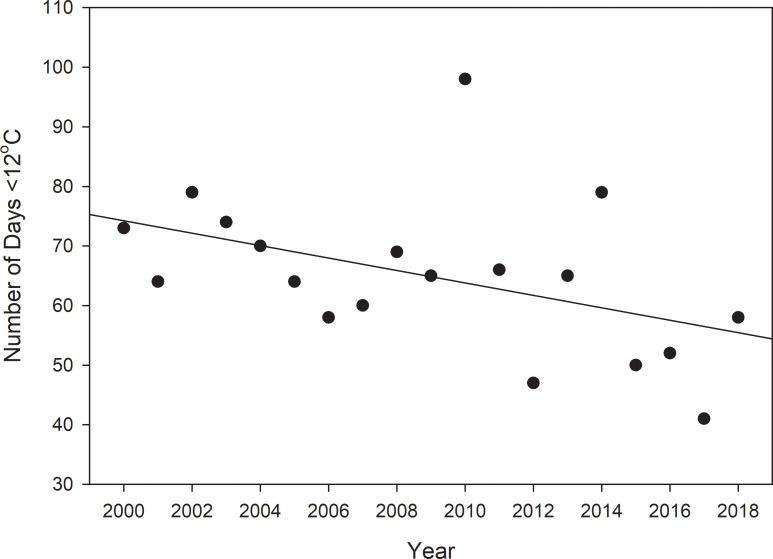
Air temperature data from the Cedar Key NOAA buoy (CDRF1) indicate rising temperatures since 2000, in terms of declining number of days <12°C.

Clear increases in air temperature were documented at the CDRF1 gauge (Fig 3). Regression analysis indicated the number of days below the lethal limit of snook has significantly (F_1,17_ = 5.72, P<0.05) declined since 2000 (Fig 3).

### Field sampling

In total, 625 snook, ranging in SL from 53 mm to 885 mm, were collected during combined fish sampling from the Suwannee River estuary. Snook were captured primarily along shorelines in open estuarine areas with the 183-m haul seine (98% of total snook catch); the 21.3-m seine and 6.1-m otter trawl comprised only 2% of the total catch. Snook captured by these two gear types were almost exclusively (93%) from tidal creek and river habitat (Table 1).

**Fig 4:**
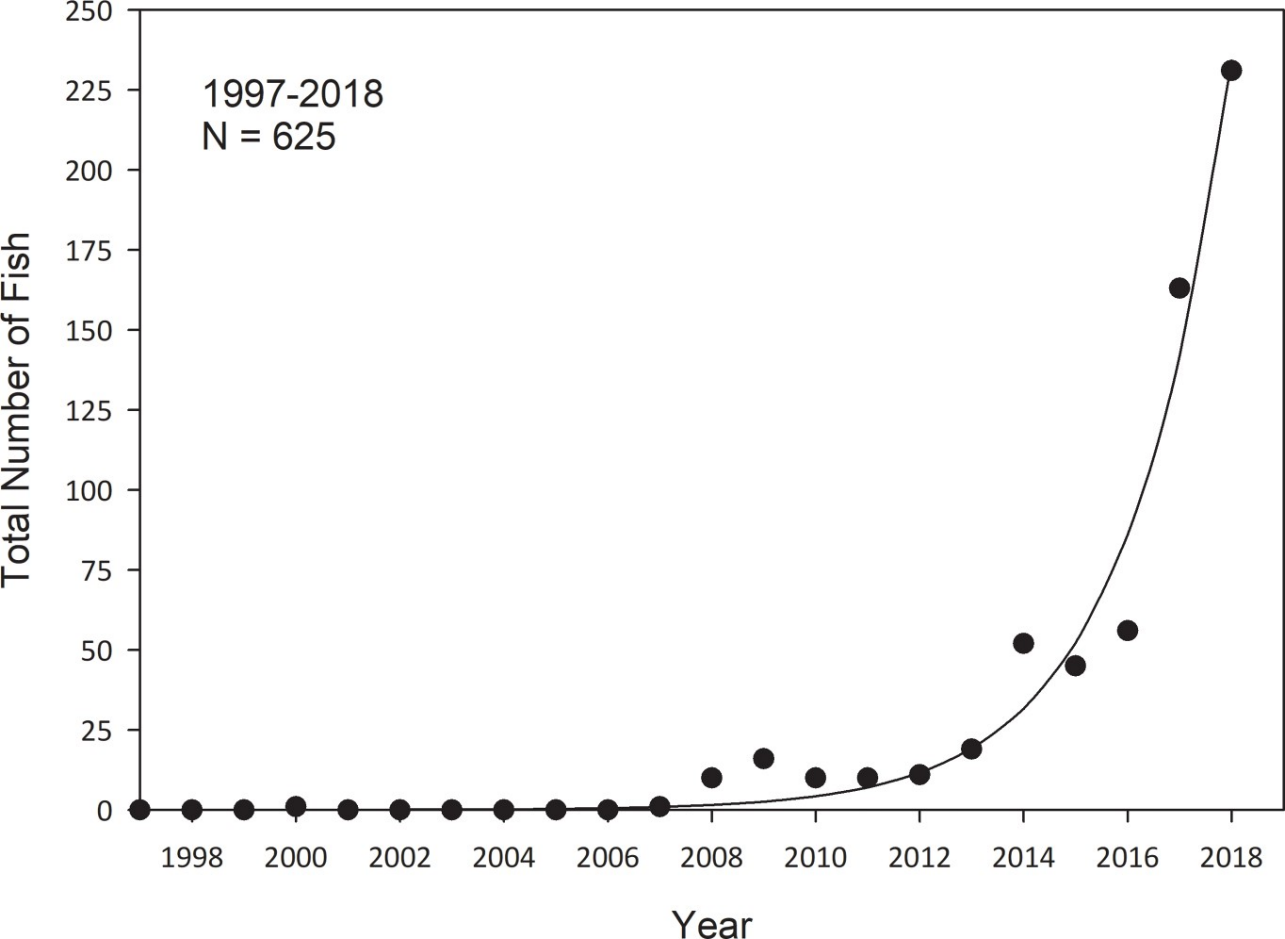
Yearly catches of snook collected in 183-m haul seines, 21.3-m seines, and 6.1-m otter trawls in the Suwannee River estuary, Florida. Data (symbols) and the fitted exponential model (line) are shown. Data represent total snook catches of all gear types combined.

**Fig 5:**
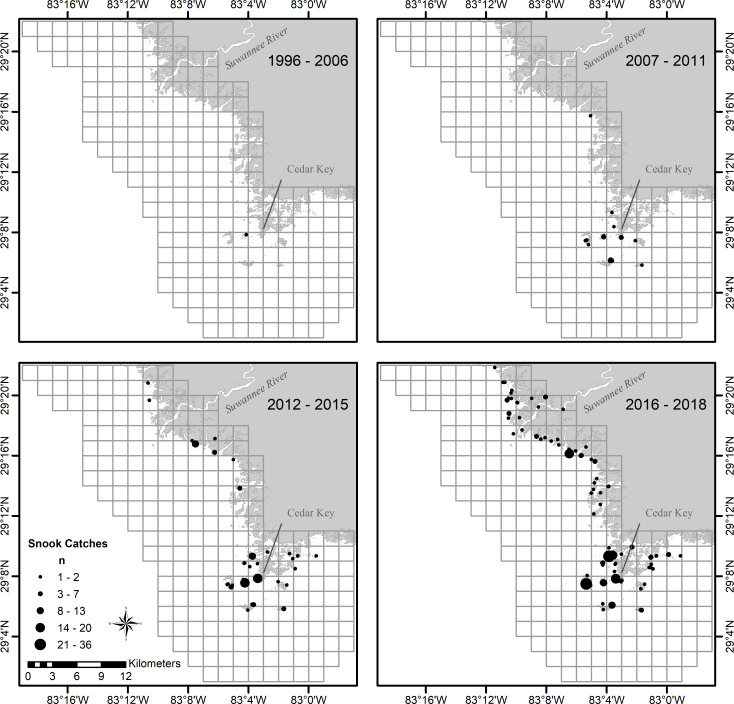
Locations of snook catches in the Suwannee River estuary, Florida, with all sampling gear combined. Panels represent blocks of data collected, 1997–2018. Grid system represents the Fisheries Independent Monitoring universe.

**Fig 6:**
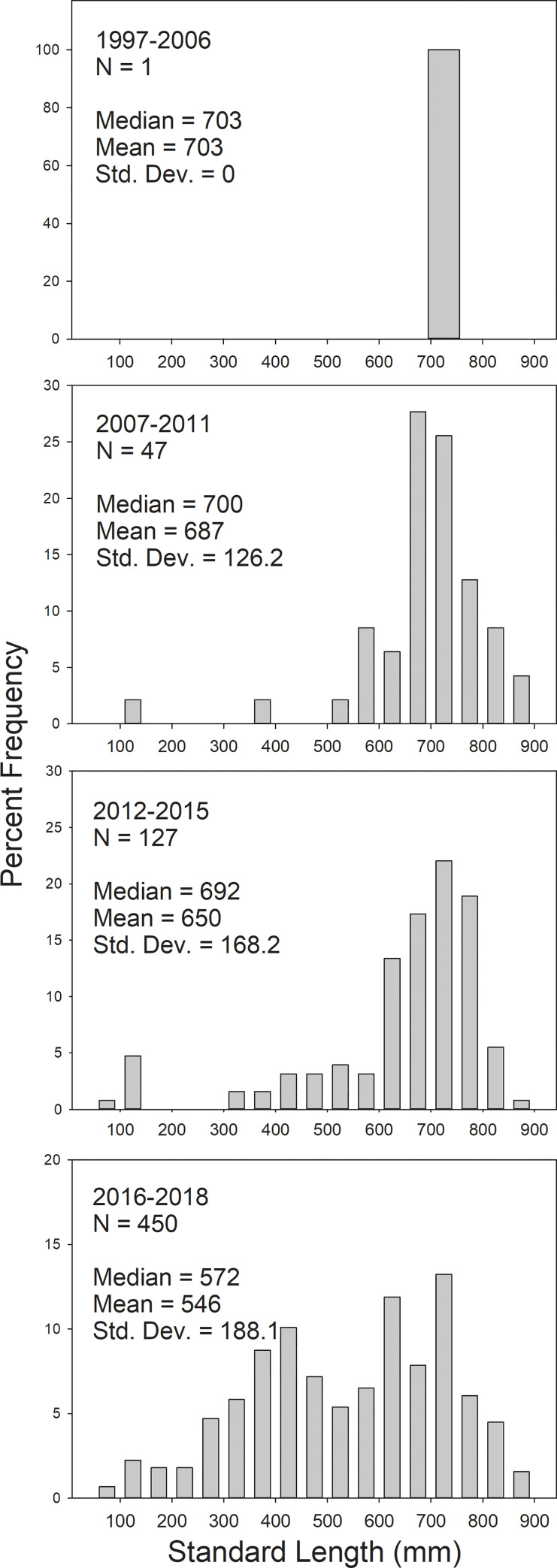
Length frequency distributions of snook collected in the Suwannee River estuary, Florida, with all sampling gear combined. Combined years align with years in Fig 5. Total number of fishes captured (N) is represented.

In 2000, the first snook (703 mm SL) was captured near the Cedar Key islands during monthly stratified random sampling (Figs 4–6), documenting the northernmost extent of its range. Another snook was not captured again until 2007, when yearly occurrences of snook began to appear during sampling. From 2012 through 2018, there was an exponential increase in snook catches. Total catches in 2017 (N = 163) tripled catches from the previous year (N = 56). In 2018, catches increased to N = 231 and was the highest of all years of sampling (Fig 4).

The exponential model provided a substantially more credible fit to the data than a constant temporal catches model. The delta AIC value for the intercept-only model was 188, indicating that the constant catch through time model had near zero credibility relative to the exponential model. Therefore, the exponential model was selected as the preferred model, indicating sharply increasing catches in recent years (Fig 4).

Expansion in spatial distribution and size composition of snook was evident through time in the Suwannee River estuary (Figs 5 and 6). During the early years of expansion, captured snook were almost exclusively larger, older individuals. We hypothesize that these individuals likely immigrated to the Suwannee River estuary from southern estuaries. Early captured (2007–2011) snook were collected primarily around the Cedar Key islands (Fig 5). During 2012–2015, the spatial distribution of snook expanded around the Cedar Key islands and to the north toward the Suwannee River. Catches of snook continued to increase and spatial distribution continued to expand into areas north of the Suwannee River in 2016–2018. Areas in which snook were captured also expanded into the river and tidal creek areas, with four snook captured in the Suwannee River and 10 captured in tidal creeks (Table 1). As catches and distribution of Snook increased during 2012–2018, the size structure changed from primarily large individuals to individuals of all sizes, including age-0 fish that had likely hatched locally. By 2018, we found a population that appeared fully established with presumed local reproduction ongoing with fish less than 100 mm SL (Fig 6).

**Fig 7:**
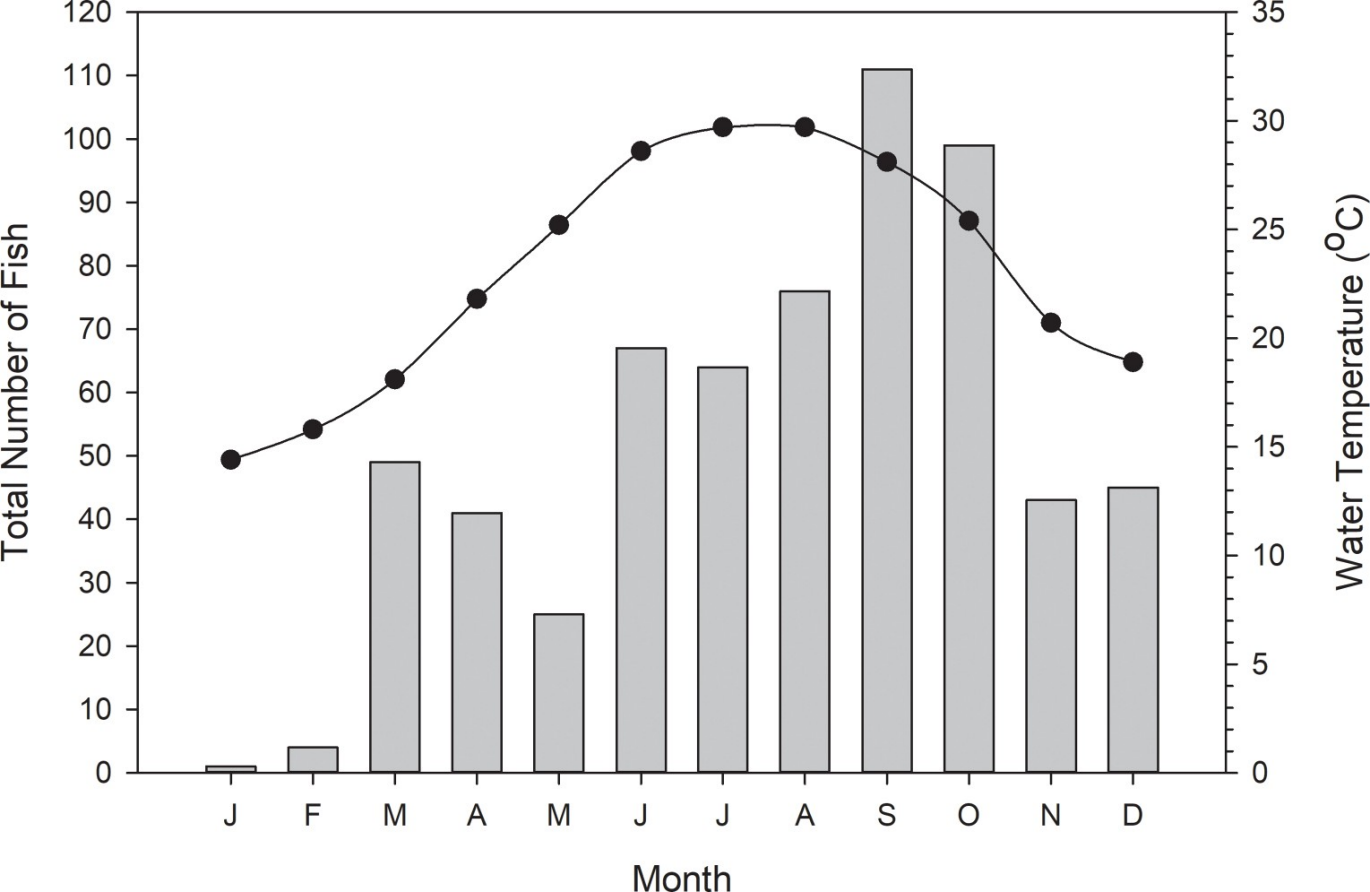
Combined monthly catches (1997–2018) of snook collected in the Suwannee River estuary, Florida, with all sampling gear combined. Monthly mean water temperatures, calculated from all sample sites during the study period whether or not a snook was caught, are represented by the black line.

Snook were captured across all months during this study but were least abundant (N = 5) during late winter (January and February), when water temperatures averaged 14.4°C and 15.8°C, respectively (Fig 7). Catches increased in March and April (N = 90) as water temperatures approached 20°C and decreased in May (N = 25). Catches continued to increase (N = 207) through the summer (June–August) as water temperatures peaked. The largest catches (N = 210) were made during a two-month period in late summer and early fall (September and October), as water temperatures started to decrease. Catches during November and December (N = 88) were similar to early spring catches, as water temperature decreased towards 20°C. We did collect snook during relatively low temperatures, including a cold event in December 2017, when water temperature dropped below 12°C. During this event, two snook were captured in water at 11.9°C, below the reported lethal low temperature of 12.5°C, and five snook were captured in water at 13.5°C. In December 2018, another 19 snook were captured in water at 13.9°C.

**Fig 8:**
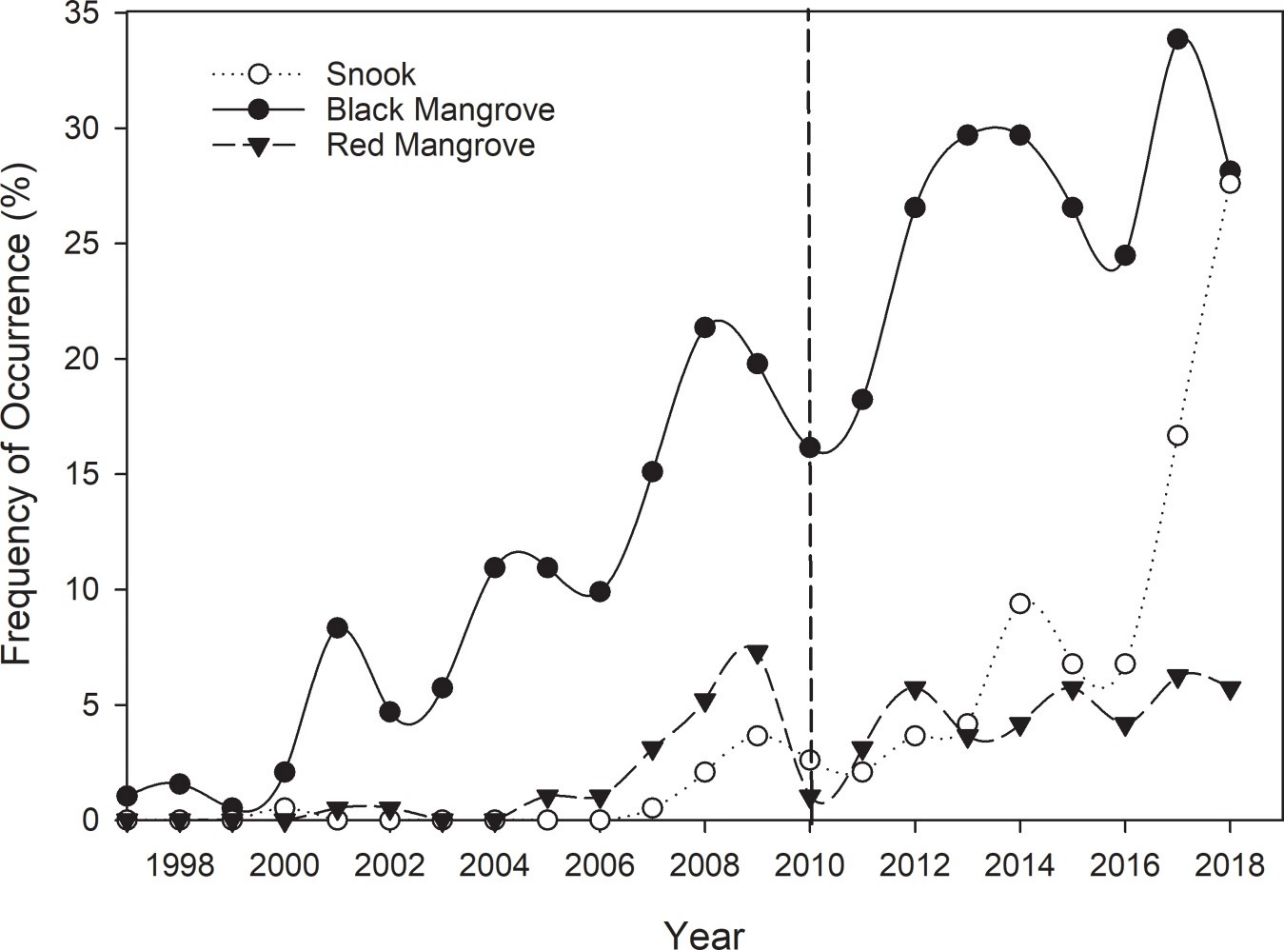
Yearly frequency of occurrence of snook, black mangrove, and red mangrove observed at 183-m haul seine sites in the Suwannee River estuary, Florida. The vertical dotted line indicates the 2010 winter freeze.

Since 2000, when the first snook was captured at its northernmost extent, there was an increase in the frequency of occurrence of black mangrove through 2018 (Fig 8). Frequency of occurrence of snook and red mangrove started to increase after 2007, as well as total snook catch per year and continued to increase throughout the study period (Figs 4 and 8). However, red mangrove, black mangrove, and snook all showed a substantial decrease in frequency of occurrence during a hard freeze in 2010, but slightly rebounded the following year and continued to increase through our study. These data support that the exponential increase in snook catches during 2016–2018 reflected an expansion in spatial distribution over time and did not result from several large catches from a few sampled areas (Figs 4 and 8).

## Discussion

The poleward expansion of snook in the northern Gulf of Mexico likely resulted from increasing water temperatures and less frequent winter freezes. The Suwannee River estuary experienced clear evidence of warming waters, in terms of temperatures above the lethal limit for snook, a trend that may have facilitated the expansion of the subtropical snook. The last extended cold event that extensively killed mangroves in this area was 1989 (author’s personal observation), and while a general trend of warming temperatures is present, it is possible that less frequent, but more intense cold events could occur via atmospheric instability in the polar regions creating stochastic events such as polar vortexes [34].

Poleward shifts in the biogeographic distributions of marine organisms have been well documented (reviewed in [1, 35,36]). These distribution changes have been documented for primary producers such as phytoplankton [37], algae [38], and emergent plants [39], and for invertebrates such as fouling organisms [40], bivalves [41], gastropods [42], squids [43], amphipods [44], and crabs [45,46]. Mobile aquatic organisms such as fishes can quickly migrate to follow optimal environmental conditions, and fish distribution patterns exhibit similar changes. For example, southern species have begun replacing northern species in California reef fish assemblages [47], and Perry et al. [48] documented similar changes in North Sea fishes, with around half of the species ranges expanding northward. In all these instances, rising temperatures have been hypothesized as the mechanism driving the range shift.

Along with poleward expansion of snook in the Gulf of Mexico (this study, [26]), there has been substantial expansion in both red mangrove and black mangrove, which provide important habitat for both juvenile and adult snook [29, 49,50]. It is unclear whether the mangrove expansion northward is a mechanism for snook expansion, but clearly the habitats are shifting from salt marsh to mangroves in this region [8, 14], which will further improve habitat for snook. Snook commonly use mangrove habitat in southern estuaries, however are habitat generalists and use habitats in proportion to their relative availability [51]. Therefore, we predict as mangroves expand northward, snook are likely to utilize those habitats.

Our observation of expanding mangrove habitat is consistent with Cavanaugh et al. [33], who reported a doubling of spatial extent of mangroves along the east coast of Florida, between latitudes 29°N and 29.75°N, which directly corresponded to our study area (29°4′ N–29°20′ N) along the west coast of Florida. Cavanaugh et al. [14] found that decreases in the frequency of cold events, rather than increases in mean air temperature, facilitated the expansion of mangroves. Daily minimum temperature has increased faster than daily maximum or mean temperature, resulting in a warming trend over the past 50 years [2, 8, 33]. Thus, the expansion of snook in this region was likely correlated with increased temperatures overall.

The sustainability of a snook population in the Suwannee River estuary may depend on the availability of thermal refugia or the ability of the species to adapt to colder climate. This newly established population is still likely to be exposed to cold events greater in magnitude or duration than events in their historical range. Snook in Florida experienced extensive mortality events during the extremely cold winters of 1989–1990, 2000–2001, and 2009–2010. Thus, we expect that snook in the Suwannee River estuary will still experience setbacks due to cold kills. The winter of 2010, for example, caused an extreme cold kill of snook that extended as far south as the Everglades and substantially altered abundance and catch by anglers [25, 52]. Recovery of snook following this cold kill took as long as four years, depending on location [53]. Snook catches in our study area were noticeably reduced by the 2010 cold kill, but fully recovered within three years.

Since 2010, winters have been relatively mild, and it is during this time that the snook population expanded substantially northward, beyond the Suwannee River. However, snook in the Suwannee River estuary survived minor cold events in 2017 and 2018, which suggests that snook at this latitude may have developed local behavioral adaptations by finding thermal refuge in the Suwannee River, tidal creeks, or areas with warm groundwater springs, common throughout the region. During these cold events in our study, snook were captured near the mouth of the Suwannee River. Movement patterns of snook can affect their vulnerability to extreme cold events [28, 53]. Snook in their historic range are known to migrate into rivers, creeks, and channels post-spawning to endure cold events [53]. Catches in our study were greatest during the presumed post-spawning season (September and October). Snook may have been congregating before migrating to the Suwannee River or tidal creek habitat to endure the winter.

A key uncertainty is the degree to which thermal refugia in the form of groundwater springs may provide resilience to this snook population and allow individuals to withstand cold kill events. We expect snook in the Suwannee River estuary to adapt a behavioral strategy that takes advantage of thermal refuges during winter; that is, they will seek groundwater springs by moving into rivers and creeks as winter sets in, returning to the open estuary as water temperatures warm again. The geology in the region is karst porous limestone with extensive springs and groundwater seeps [54]. Natural springs provide constant water temperature throughout the year, and, because spring water is warmer than ambient water temperature in winter, the coastal springs provide thermal refugia for cold-intolerant fishes and marine mammals [55]. Groundwater flow rates are strongly influenced by precipitation patterns and water levels in the Lower Suwannee River [56,57]. Thus, changes in freshwater flow patterns could influence the availability of thermal refugia during winter, and understanding this relationship is a key future research need.

Research is also needed to determine whether the biology of snook differs at a more northern latitude, particularly regarding differences in spawning and growth, which may influence the persistence of snook and its impact on fishery management strategies. Cold weather in northern latitudes begins earlier and ends later than in areas farther south. Therefore, we expect the snook spawning season, which has been correlated to water temperature during summer months in southern latitudes [58,59], to be shorter in more northern areas. A species at the northern extent of its range also tends to grow faster than those at lower latitudes. This is thought to be a compensatory response to a shorter growing season, known as the counter gradient hypothesis [60]; when the species has the opportunity to grow, it does so quickly before cold temperatures slow their growth again [61]. Thus, we expect snook growth rates in the Suwannee River estuary to be faster than in their historic range. Similarly, fish natural mortality is affected by both growth rate and temperature [62], and future research should explore whether growth and mortality differ in the northern expansion of the snook’s range, which could alter optimal management plans (e.g., bag or size limits).

We found evidence for local reproduction of snook, with age-0 fish first occurring in 2010 followed by juvenile fish captured in subsequent years. The shoreline of the Suwannee River estuary is characterized by an expansive network of coastal tidal creeks that provide ideal habitat for age-0 and juvenile snook. Beginning in 2016, age-0 and juvenile snook were commonly observed within tidal creeks, further suggesting self-recruitment. However, the variability of recruitment and level of survival are not clear and warrant further investigation. Additional evidence of local reproduction is that island habitats and channels around Cedar Key are consistent with descriptions of spawning sites for snook farther south [59]. Summer water temperatures are warm enough for spawning at the Cedar Keys, which in southern estuaries typically begins in April or May, when water temperatures reach approximately 22°C [63,64]. In our study, catches decreased at that time, which may have been attributed to snook moving to potential spawning aggregations, such as deep channels around barrier islands or offshore wrecks and reefs, where they were not vulnerable to our sampling gear [59].

Identification of snook nursery habitat should be a high priority if resource managers wish to encourage a sustainable recreational snook fishery in the Suwannee River estuary. Age-0 snook typically recruit to areas well into the land margin such as river backwaters, the headwaters of tidal creeks and high-marsh ponds [65–67]. These habitats often lie at the interface with coastal development and urbanization and so are particularly vulnerable to anthropogenic impacts. Quantifying the habitat use of coastal wetlands by a recreationally important species may help raise awareness of their value. Possible nursery habitats can be identified by expanding fisheries monitoring into remote tidal creeks and marsh ponds, where snook are known to reside. Then it may be possible to assess the relative importance of each nursery (those that support the highest growth rates and condition [68,69]) and its contribution to the adult population [70].

Changes in species distributions could have trophic impacts for historically native predators and prey fish populations [1]. Snook support important fisheries and are prized by anglers, but its spatial expansion and the resulting competition for resources could negatively impact historically dominant inshore sport fish in the northern Gulf, particularly red drum *Sciaenops ocellatus* and spotted seatrout *Cynoscion nebulosus*. Snook exhibit a high degree of diet overlap with red drum in South Florida, where both species historically occur [71]. Red drum, snook, and spotted seatrout are all mid-trophic-level predators that consume a range of demersal fishes and invertebrates [71,72]. Red drum populations increased sharply in South Florida following an extreme statewide cold kill of snook in 2010, suggesting that release from competition with or predation by snook could have allowed an increase in red drum abundance [52]. The expansion of snook into the Suwannee River estuary could influence abundance of prey populations and growth rates for red drum and spotted seatrout, ultimately influencing sustainable harvest strategies for these species. Thus, future research should explore habitat and diet overlap among historically native and novel predators, as well as prey population responses to expanding predator populations like snook.
